# Human First, Machine Second: Perspectives From the Age 85+ on Artificial Intelligence

**DOI:** 10.1093/geroni/igaf122.1370

**Published:** 2025-12-31

**Authors:** Taylor Brennan, Niels Wu, Sophia Ashebir, Lisa D’Ambrosio

**Affiliations:** MIT, Boston College, Cambridge, Massachusetts, United States; Massachusetts Institute of Technology, Cambridge, Massachusetts, United States; Massachusetts Institute of Technology, Cambridge, Massachusetts, United States; Massachusetts Institute of Technology, Cambridge, Massachusetts, United States

## Abstract

Rapid advancements in the capabilities of artificial intelligence (AI) have sparked interest in its potential to support an aging population. Minimal work has examined how the oldest of older adults engage with and think about AI in their lives, despite this demographic being one that might especially benefit from AI technologies. Leveraging data from focus groups (N = 14) and a questionnaire (N = 25), this study examines a sample of the age 85+ and their attitudes toward, experiences with, and trust in AI. Participants were recruited from the MIT AgeLab’s 85+ Lifestyle Leaders Panel, a longitudinal study of octogenarians and nonagenarians. Initial findings indicate participants had low familiarity and experience with AI; greatest levels of familiarity were around AI-powered voice assistants. While participants expressed limited experience, many showed openness to learning about AI’s potential to support health and care management, cognition and memory, technology troubleshooting, and personal security. Participants preferred AI that enhanced rather than replaced human capabilities. Their reported concerns about AI centered on its surveillance capabilities, data security, and potential to reinforce social biases, particularly ageism. These findings underscore opportunities to better educate the age 85+ about AI, the advantages of involving them in AI design, and the need to consider how AI may contribute to or bridge digital divides within this population.

